# B81 Bone Vibrator-Induced Vestibular-Evoked Myogenic Potentials: Normal Values and the Effect of Age

**DOI:** 10.3389/fneur.2022.881682

**Published:** 2022-05-11

**Authors:** Yuzhong Zhang, Zichen Chen, Huandi Zhao, Jiali Shen, Bo Zhong, Qiong Wu, Jun Yang, Yulian Jin, Qing Zhang, Pengyu Ren

**Affiliations:** ^1^Department of Otorhinolaryngology, Head and Neck Surgery, Second Affiliated Hospital of Xi'an Jiaotong University, Xi'an, China; ^2^Department of Otorhinolaryngology, Head and Neck Surgery, Xinhua Hospital, Shanghai Jiao Tong University School of Medicine, Shanghai, China; ^3^Institute of Ear Science, School of Medicine, Shanghai Jiao Tong University, Shanghai, China; ^4^Shanghai Key Laboratory of Otolaryngology and Translational Medicine, Shanghai, China; ^5^Division of Mechanics and Acoustics, National Institute of Metrology, Beijing, China

**Keywords:** B81 bone vibrator, aging, cervical vestibular-evoked myogenic potential, ocular vestibular-evoked myogenic potential, vestibular function

## Abstract

**Objective:**

To define the normal values and examine the influence of aging on B81 bone vibrator-induced cervical vestibular-evoked myogenic potentials (B81-cVEMPs) and ocular vestibular-evoked myogenic potentials (B81-oVEMPs).

**Methods:**

Seventy healthy subjects, divided into seven groups according to their ages, were enrolled in this study. The 4–9-, 10–19-, 20–29-, 30–39-, 40–49-, 50–59-, and 60–70-year-old participants were divided into groups I–VII, respectively. B81-cVEMP and B81-oVEMP were recorded in each group.

**Results:**

The B81-cVEMP response rates for groups I–VII were 100, 100, 100, 100, 95, 95, and 75%, respectively, with significant differences only between groups I–VI and group VII (*p* = 0.047, *p* < 0.05). The B81-oVEMP response rates for groups I–VII were 100, 100, 100, 100, 70, 65, and 40%, respectively, with significant differences only between groups I–IV and groups V–VII (*p* = 0.020, *p* = 0.008, *p* = 0.000; *p* < 0.05). The threshold, P13, and N23 latencies of B81-cVEMP positively correlated with age (r = 0.756, *p* = 0.000; r = 0.357, *p* = 0.003; r = 0.316, *p* = 0.009; *p* < 0.05). The raw amplitudes and corrected amplitudes negatively correlated with age (r = −0.641, *p* = 0.000; r = −0.609, *p* = 0.000, *p* < 0.05). For B81-oVEMP, the corrected amplitudes negatively correlated with age (r = −0.638, *p* = 0.000, *p*<0.05), but the threshold and N10 latency positively correlated with age (r = 0.768, *p* = 0.000; r = 0.334, *p* = 0.009, *p* < 0.05). Moreover, the interaural asymmetry ratio did not significantly correlate with age for B81-cVEMP and B81-oVEMP.

**Conclusion:**

As age increased, the B81-cVEMP response rate decreased, the thresholds increased, P13 and N23 latencies were prolonged, and the raw amplitude and corrected amplitude decreased. The B81-oVEMP response rate and corrected amplitude decreased, the thresholds increased, and N10 latency was prolonged with age. These changes are probably due to the occurrence of morphological and functional changes in the vestibular system with aging. Therefore, we suggest establishing different reference values according to different age groups when evaluating the VEMP results in patients with vestibular diseases.

## Introduction

The vestibular-evoked myogenic potential (VEMP) is a non-invasive, objective, and easily accepted clinical test that is now widely used to assess otolithic function (1–3). The potentials recorded on the surface of the sternocleidomastoid (SCM) muscles are called cervical VEMPs (cVEMPs), and those recorded on the surface of the extraocular muscles are called ocular VEMPs (oVEMPs) (4–7). Therefore, cVEMP is a manifestation of the vestibulocollic reflex and predominantly reflects saccular function, while oVEMP is a manifestation of the vestibulo-ocular reflex (VOR) and reflects utricular function (1, 3, 8).

Currently, there are three types of stimulation used to detect VEMPs: air-conducted sound (ACS), bone-conducted vibration (BCV), and galvanic vestibular stimulation (2, 3, 8, 9). VEMP elicited by ACS stimulation is widely used. However, there are some drawbacks to the use of ACS as a stimulus delivery method for VEMPs. First, high-intensity ACS stimuli increase hazardous sound exposure when the equivalent ear canal volumes are less than or equal to 0.8 ml. Additionally, patients with conductive hearing loss or air–bone gaps may not generate VEMPs in response to ACS (10). BCV is a viable alternative mode of stimulation for eliciting VEMP responses. Various bone vibrators have been used to elicit VEMPs, such as tap hammer (9, 11), mini-shaker 4810 (3, 12), B-70B (13), V201 (14), Radioear B71 (6, 15), and Radioear B81 (16, 17). Radioear B71 and B81 are universal components for the testing of hearing levels using the bone conduction method. It has been reported that relative to the B71, the B81 vibrator has higher maximum output levels and lower distortion to be used in pure-tone audiometry (18). However, Clinard et al. (16) reported that the maximum output levels of the B81 vibrator were equivalent to B71 at 500 Hz (stimuli were tone bursts with a fixed duration of 8 ms). Romero et al. (19) used the same frequency and duration of stimuli (500 Hz, rise-plateau-fall times: 4-0-4 ms), as Clinard et al. showed no statistically significant differences between the amplitudes of the B71- and B81-cVEMP. Hence, whether the B81 vibrator can be effectively used for c/oVEMP response requires further study.

Age-related morphological changes may occur in the vestibular system from the end organs to the central nuclei, including loss of hair cells, decreased numbers of vestibular nerve cells, and loss of neurons in the vestibular nucleus (20–23). Therefore, the effects of advancing age on the response rate, latency, and amplitude of ACS-VEMPs have been well explored (24, 25). However, the age-related impact on the B81-VEMPs remains unclear. Therefore, this study aimed to establish normal values and examine the effect of age on c- and oVEMP responses using the B81 vibrator.

## Materials and Methods

### Subjects

Healthy subjects with no hearing or vestibular deficits were recruited from the Second Affiliated Hospital of Xi'an Jiaotong University. Pure tone audiometry, acoustic immittance, and otoscope tests were performed on all subjects to exclude those with hidden hearing loss (pure tone average threshold ≥ 20 dB), asymmetric hearing loss, an air–bone gap larger than 10 dB, and abnormal tympanic pressure. Finally, 70 subjects ranging from 4 to 70 years were divided into seven groups by decade. Each group consisted of 10 subjects (five men and five women). Group I included subjects aged 4–9 years, Group II subjects aged 10–19 years, Group III subjects aged 20–29 years, Group IV subjects aged 30–39 years, Group V subjects aged 40–49 years, Group VI subjects aged 50–59 years, and Group VII subjects aged 60–70 years. The study protocol was reviewed and approved by Xi'an Jiaotong University (approval number: 2016205). All participants were informed and volunteered to participate in this study.

### BCV Stimuli and Recording Parameters

**Figure 1:**
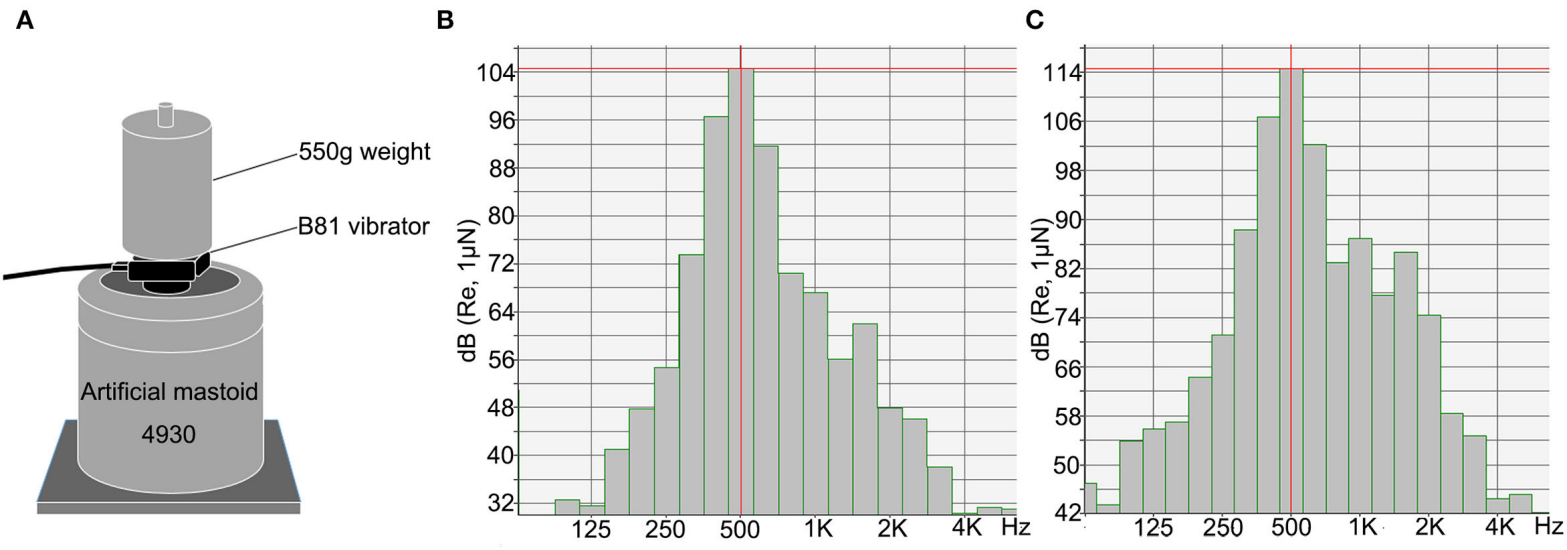
The Radioear B81 bone vibrator was calibrated using an artificial mastoid. **(A)** Artificial mastoid 4930 (Brüel & Kjær, Denmark) was used to calibrate the B81 bone vibrator. A constant force was maintained by adding a 550 g weight to the artificial mastoid when calibrating. **(B,C)** The spectra show energy distribution of 500 Hz tone burst of the B81 bone vibrator at the output of 119.5 dB peFL (stimuli were delivered at 50 dB nHL) **(B)** and 129.5 dB peFL (stimuli were delivered at 60 dB nHL) **(C)**. The central energy is at 500 Hz.

The cVEMP and oVEMP tests were performed using the Interacoustics Eclipse system (Interacoustics, Middelfart, Denmark). BCV stimuli were delivered using a Radioear B81 bone vibrator (housing dimensions: height, 16 mm; length, 31.7 mm; width, 18.2 mm; weight, 20 g). The stimulus level was measured in decibels of peak force level (dB peFL) (re: 1 μN) using an artificial mastoid 4930 (Brüel & Kjær, Denmark) (Figure 1). Stimuli with the alternating polarity of 500 Hz tone bursts with a fixed duration of 6 ms (rise/fall time: 2 ms, plateau time: 2 ms) were delivered. For subjects ≥20 years, the stimulation started from a maximum intensity of 134.5 dB peFL (5.3 N), and for subjects <20 years, the stimulation intensity started from 129.5 dB peFL (3.0 N). The stimulation rate was 5.1/s, and 80 responses were averaged for each run. The electromyogram (EMG) signals were amplified and bandpass filtered between 10 and 1,000 Hz. The recording window was 0 ms to 80 ms. To measure background muscle activity for cVEMP, subjects were provided feedback on the level of activity in their SCM muscles during data collection. They were required to maintain a background muscle activity of 50–200 μV. Electrode impedance was maintained below 5 kΩ.

### B81-cVEMP and B81-oVEMP Recording

**Figure 2:**
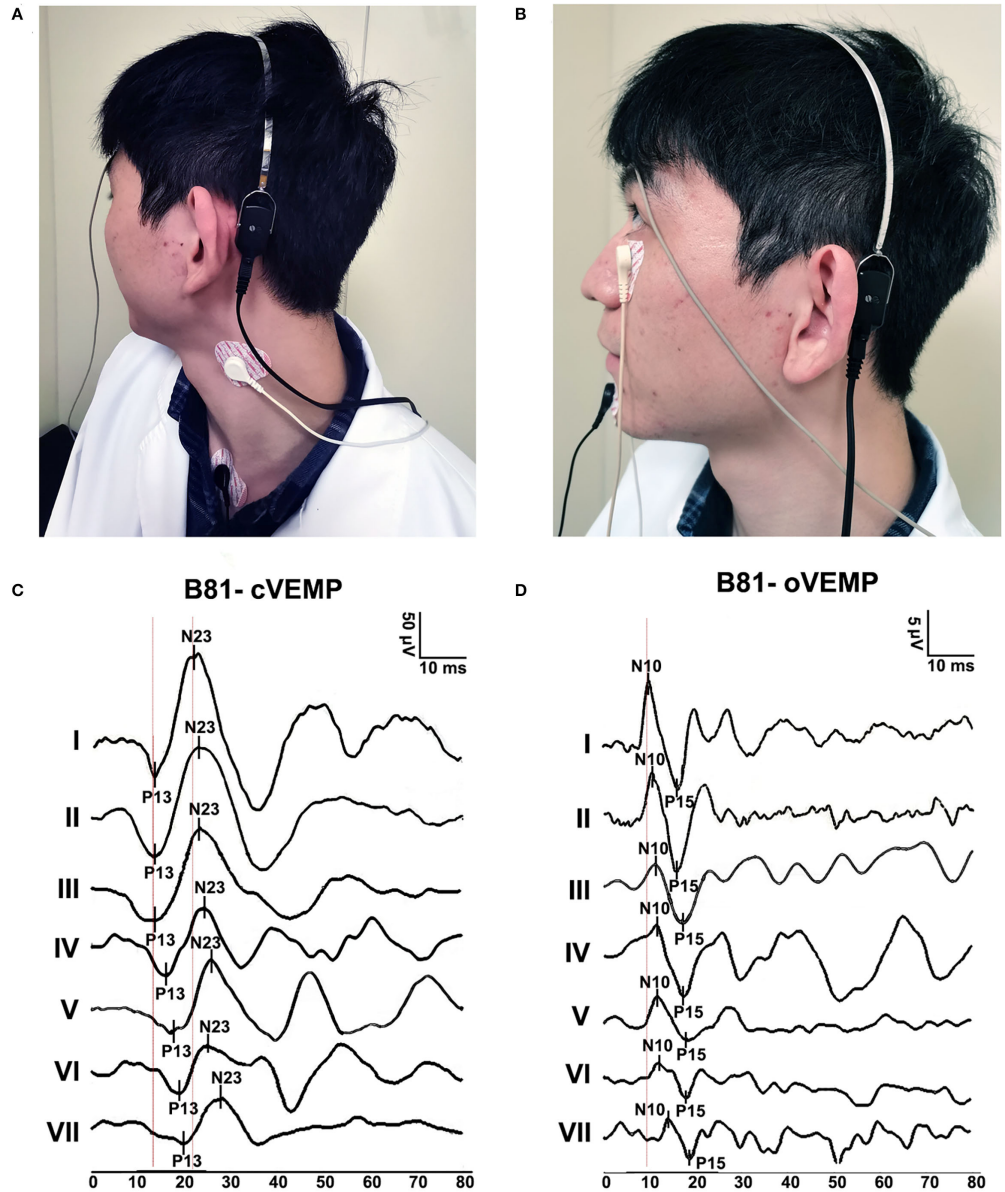
B81-cVEMP and B81-oVEMP test positions and waveforms. **(A)** Testing position of B81-cVEMP. **(B)** The test position of B81-oVEMP. **(C)** Typical B81-cVEMP waveforms from I to VII age groups. The P13 and N23 latencies were prolonged, and the raw amplitude decreased with aging. **(D)** Typical B81-oVEMP waveforms from groups I to VII. The N10 latency of B81-oVEMP was prolonged with age.

The subjects remained in a sitting position. The B81 bone vibrator was placed on the mastoid (3 cm behind and 2 cm above the external acoustic meatus) on the stimulation side (Figures 2A,B) (15).

The cVEMP recording electrodes were positioned on the upper half of the bilateral SCM muscles, with a common reference electrode placed on the upper sternum. One ground electrode was placed in the middle of the forehead (Figure 2A). The subject sat with the head rotating sideways toward one shoulder to activate the SCM muscles for the recording duration (26).

The oVEMP recording electrodes were placed on the face inferior to each eye, approximately 1 cm below the center of the lower eyelid. A common reference electrode was positioned on the jaw. One ground electrode was placed in the middle of the forehead (Figure 2B). During recording, the subject was instructed to look upward, with a vertical visual angle of ~30–35° above the horizon (14).

When a typical cVEMP and oVEMP waveform (Figures 2C,D) appeared, it was decremented by 10 dB each time until the waveform disappeared or the reproducibility was poor, and then increased by 5 dB until the waveform appeared. The threshold for B81-VEMPs was the lowest level in dB peFL at which VEMPs could be reliably recorded. The initial peak-to-peak amplitude indicated the raw amplitude. To compensate for uneven SCM muscle contractions, raw amplitudes were divided by the mean rectified EMG activity during the 20 ms before the onset of the stimulus (corrected amplitudes) (27). The raw interaural asymmetry ratio (IAR) was calculated as IAR =100 (larger amplitude – smaller amplitude) / larger amplitude + smaller amplitude). The corrected IAR was calculated based on the corrected amplitudes. Subjects with unilaterally or bilaterally absent responses were not included in the IAR calculation. For B81-oVEMP, the corrected amplitudes and corrected IAR were calculated in the same way as B81-cVEMP. The threshold, P13(N10) latency, N23(P15) latency, P13–N23 (N10-P15) interval latency, raw amplitudes, raw IAR, corrected amplitudes, and corrected IAR of the maximum stimulation intensity (≥ 20 years old: 134.5 dB peFL, <20 years old: 129.5 dB peFL) were measured.

### Statistical Analyses

All the statistical analyses were performed using SPSS 22.0 (IBM, Armonk, NY). The chi-square test or Fisher's exact test was used to compare the response rate of B81-VEMPs between each age group (categorical variables). Kruskal–Wallis one-way analysis of variance was performed for the multiple comparisons of P13 (N10) latency, N23 (P15) latency, P13- N23 (N10-P15) interval latency, amplitudes, and IAR (continuous variables). Bonferroni (homogeneity of variance) or Tamhane's (heterogeneity of variance) adjusted multiple comparisons were used as *post-hoc* tests. Spearman's correlation analyses were used to determine the relationship between age and B81-VEMP parameters. Using Spearman's correlation analysis, linear regression curves were computed only when a statistically significant correlation was observed. The curves were constructed with Prism 8.0 (GraphPad, La Jolla, CA). For all comparisons, *p* < 0.05 were considered statistically significant.

## Results

### Response Rate of B81-cVEMP

The response rate of B81-cVEMP in all subjects was 95% (133/140). The rate was 100% for groups I–IV, 95% for groups V and VI, and 75% for group VII. There were statistically significant differences among the age groups (*p* = 0.026, *p* < 0.05, chi-square test; Table 1). Further analyses showed statistically significant differences between groups I–IV and VII (*p* = 0.047, *p* < 0.05, Fisher's exact test; Table 1). There was no statistically significant difference between the other groups.

**Table 1:** The response rate and threshold of B81-cVEMP with increasing age.

**Age groups**	**Response rate**	**Threshold (dB peFL)**		
		**Mean ±SD**	**Median**	**IQR**
I	100% (20/20)	111.75 ± 5.50	114.50	104.50–114.50
II	100% (20/20)	113.75 ± 5.45	114.50	109.50–119.50
III	100% (20/20)	112.50 ± 3.77	114.50	109.50–114.50
IV	100% (20/20)	121.25 ± 6.34	119.50^b^	115.75–124.50
V	95% (19/20)	125.55 ± 4.88	124.50^b^	124.50–129.50
VI	95% (19/20)	125.55 ± 3.15	124.50^b^	124.50–129.50
VII	75% (15/20)^a^	129.83 ± 3.52	129.50^b, c, d^	129.50–134.50
*p*-value	0.026	/	0.000	/

*p-value, chi-square test and Kruskal–Wallis one-way analysis of variance; ^a^p < 0.05, Fisher's exact test, compared with groups I–IV; ^b^p < 0.01, compared with groups I–III; ^c^p < 0.01, compared with group IV; ^d^p < 0.05, compared with group VI (Tamhane-adjusted t-test). B81-cVEMP, B81 bone vibrator-induced cervical vestibular-evoked myogenic potential; IQR, interquartile range; peFL, peak force level; SD, Standard deviation*.

### Threshold of B81-cVEMP

**Figure 3:**
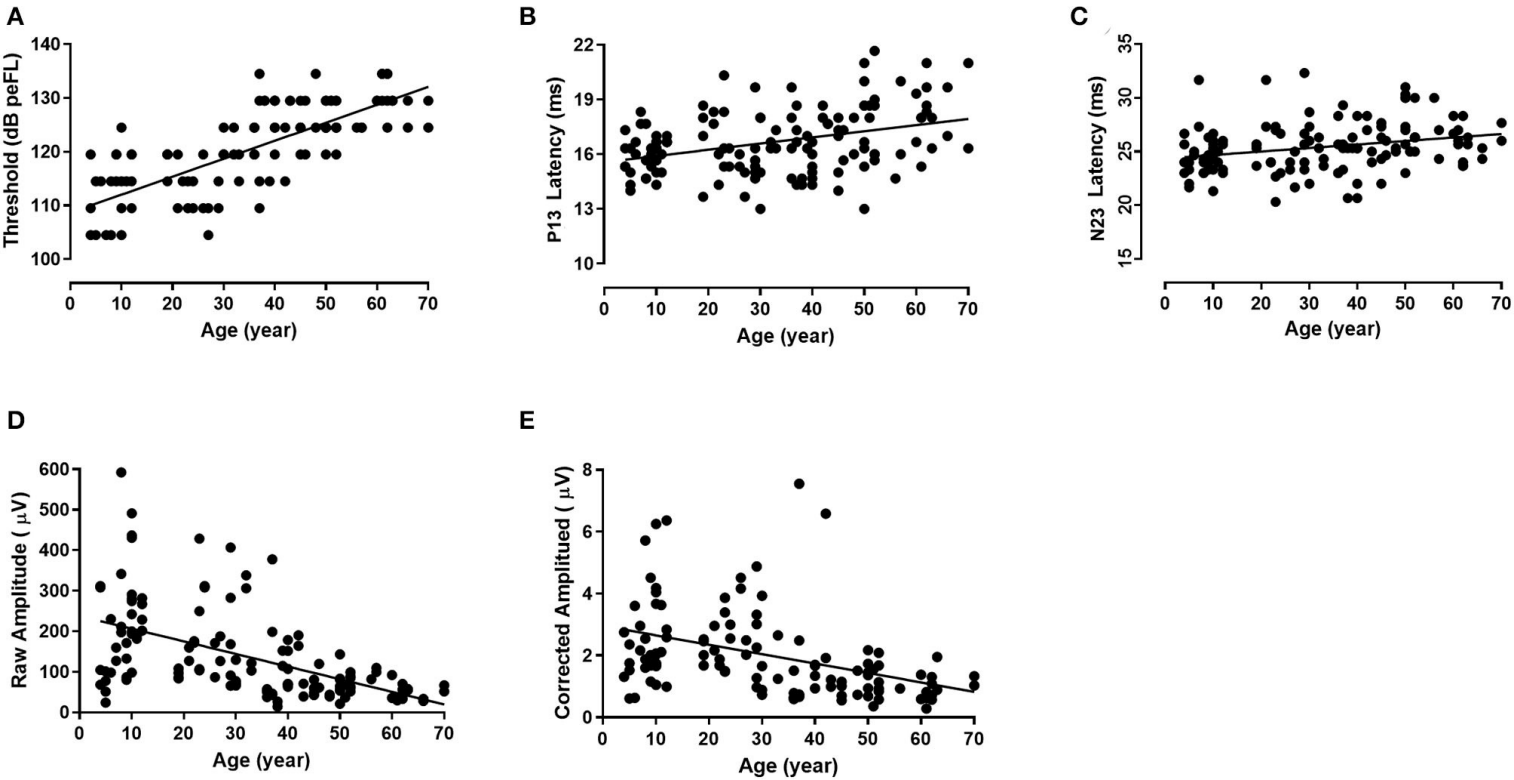
The relationship between age and individual B81-cVEMP parameters (linear regression). The threshold (r^2^ = 0.571, *p* = 0.000, *p* < 0.01) **(A)**, P13 latency (r^2^ = 0.143, *p* = 0.001, *p* < 0.01) **(B)**, and N23 latency (r^2^ = 0.096, *p* = 0.010, *p* < 0.05) **(C)** are positively correlated with age, whereas the raw amplitude (r^2^ = 0.288, *p* = 0.000, *p* < 0.01) **(D)** and corrected amplitude (r^2^ = 0.246, *p* = 0.000, *p* < 0.01) **(E)** are negatively correlated with age.

The median of the threshold of B81-cVEMP for groups I–VII was 114.50, 114.50, 114.50, 119.50, 124.50, 124.50, and 129.50 dB peFL, respectively, with significant differences in the threshold between groups I–III and IV–VII (*p* = 0.000, *p* < 0.01, Tamhane-adjusted *t*-test; Table 1). In addition, there was also a significant difference for group VII compared to groups IV and VI (*p* = 0.000, *p* < 0.05; *p* = 0.019, *p* < 0.05, Tamhane-adjusted *t*-test; Table 1). The B81-cVEMP threshold showed an increase with age (r = 0.756, *p* < 0.01, Spearman's correlation analysis). Figure 3A shows that the linear regression curve depicts the relationship between age and the B81-cVEMP threshold (y = 0.3344x + 108.7).

### P13 and N23 Latencies and P13-N23 Interval of B81-cVEMP

The mean, standard deviation, median, and interquartile range (IQR) of the descriptive statistics across ages are summarized in Table 2. The Kruskal–Wallis test revealed significant differences in the P13 latency (*p* = 0.003, *p* < 0.01) and N23 latency (*p* = 0.008, *p* < 0.01) of B81-cVEMP among the seven groups (Table 2). For the P13 latency of B81-cVEMP, there were statistically significant differences between groups I–III and VII (*p* = 0.001, *p* = 0.018, *p* = 0.012, *p* < 0.05, Tamhane-adjusted *t*-test; Table 2). There was no statistically significant difference between groups I–VI. The N23 latency of B81-cVEMP was significantly different between group VI and groups I and II (*p* = 0.004, *p* = 0.035, *p* < 0.05, Bonferroni-adjusted *t*-test; Table 2); there was no significant difference between the other groups. For the P13-N23 interval of B81-cVEMP, there were no statistically significant differences between groups I–VII (*p* = 0.390, *p* > 0.05, Kruskal–Wallis test; Table 2). Spearman's correlation analysis revealed that significant correlations existed between the age factor and the P13 latency (r = 0.357, *p* = 0.003, *p* < 0.01) and N23 latency (r = 0.316, *p* = 0.009, *p* < 0.01), but not with the P13–N23 interval (r = −0.017, *p* = 0.839, *p* > 0.05). The P13 and N23 latencies of B81-cVEMP waveforms from I to VII groups are shown in Figure 2C. Figure 3B shows the relationship between age and the P13 latency of B81-cVEMP using a linear regression curve (y = 0.03388x + 15.57). Figure 3C shows the relationship between age and the N23 latency of B81-cVEMP (y = 0.03264x + 24.36).

**Table 2:** The latencies and P13-N23 interval of B81-cVEMP with increasing age.

**Age groups**	**P13 latency (ms)**			**N23 latency (ms)**			**P13-N23 interval (ms)**		
	**Mean ±SD**	**Median**	**IQR**	**Mean ±SD**	**Median**	**IQR**	**Mean ±SD**	**Median**	**IQR**
I	15.97 ± 1.18	16.00	15.08–16.59	24.58 ± 2.22	24.00	23.08–25.50	8.62 ± 1.59	8.34	7.67–9.24
II	16.27 ± 1.16	16.50	15.67–16.92	24.73 ± 1.29	25.00	23.75–25.67	8.47 ± 1.45	8.84	7.08–9.33
III	16.24 ± 1.76	15.84	15.08–17.34	25.28 ± 2.99	24.50	23.33–26.91	9.03 ± 2.09	8.84	8.00–10.26
IV	16.43 ± 1.68	16.50	14.75–17.83	25.25 ± 2.23	25.33	23.42–26.33	8.82 ± 1.97	9.00	8.00–10.42
V	16.44 ± 1.44	16.67	15.00–17.67	25.26 ± 2.09	25.33	24.33–27.00	8.82 ± 1.70	9.00	7.33–9.67
VI	17.58 ± 2.30	18.00	16.00–19.00	27.16 ± 2.37	26.33^b^	25.00–30.00	9.58 ± 2.72	10.00	7.00–11.33
VII	18.22 ± 1.70	18.00^a^	16.67–19.67	25.91 ± 1.54	26.00	24.33–27.00	7.69 ± 2.11	7.67	5.67–9.67
*p*-value	/	0.003	/	/	0.008	/	/	0.390	/

*p-value, Kruskal–Wallis one-way analysis of variance. ^a^p < 0.05, compared with groups I–III (Tamhane-adjusted t-test); ^b^p < 0.05, compared with groups I and II (Bonferroni-adjusted t-test); B81-cVEMP, B81 bone vibrator-induced cervical vestibular-evoked myogenic potential; IQR, interquartile range; SD, Standard deviation*.

### Raw Amplitudes, Corrected Amplitudes, Raw IAR, and Corrected IAR of B81-cVEMP

There was a statistically significant difference both in the raw amplitudes and corrected amplitudes of B81-cVEMP (*p* = 0.000, *p* < 0.01, Kruskal–Wallis test; Table 3). No significant differences were found for the raw IAR among the seven groups (*p* = 0.919, *p* > 0.05, Table 3), while the corrected IAR was statistically significantly different (*p* = 0.015, *p* < 0.05; Table 3). The IAR of group VII was larger than group III. The Tamhane-adjusted *t*-test showed no significant change in amplitude up to group III, whereas a significant reduction in peak-to-peak amplitude was observed beyond this group. Using Spearman's analysis, both the raw and corrected amplitudes showed a significant negative correlation with age (r = −0.641, *p* = 0.000, *p* < 0.01; r = −0.609, *p* = 0.000, *p* < 0.01). The raw and corrected IAR showed no correlation with age (r = −0.089, *p* = 0.483, *p* > 0.05; r = 0.072, *p* = 0.601, *p* > 0.05). Figure 2C shows the raw amplitudes of B81-cVEMP gradually decrease from I to VII age groups. Figures 3D,E show an inverse relationship between age and the raw/corrected amplitude of B81-cVEMP (y = −3.116x + 237.7; y = −0.03044x + 2.956).

**Table 3:** Raw amplitudes, corrected amplitudes, raw IAR, and corrected IAR of B81-cVEMP with increasing age.

**Age groups**	**Raw amplitudes (μV)**			**Corrected amplitudes**			**Raw IAR (%)**			**Corrected IAR (%)**		
	**Mean ±SD**	**Median**	**IQR**	**Mean±SD**	**Median**	**IQR**	**Mean±SD**	**Median**	**IQR**	**Mean±SD**	**Median**	**IQR**
I	173.85 ± 133.61	130.05	81.94-225.42	2.26 ± 1.30	1.94	1.46–2.81	21.00 ± 13.62	23.00	9.00–33.75	34.74 ± 20.08	35.00	15.50–54.50
II	233.13 ± 117.57	215.20	126.68–281.28	2.79 ± 1.50	2.29	1.81–3.66	17.40 ± 14.62	13.50	5.00–27.25	27.30 ± 13.91	29.00	17.50–36.00
III	191.09 ± 106.29	169.80	104.38–274.28	2.67 ± 1.11	2.52	1.73–3.37	16.40 ± 10.96	17.00	7.75–26.75	9.80 ± 5.65	9.00	5.50–14.50
IV	119.39 ± 106.19	74.33^a^	47.35–146.63	1.86 ± 1.91	1.06^e^	0.71–2.52	14.40 ± 10.97	11.00	4.75–26.25	35.39 ± 22.27	36.00	10.00–51.00
V	86.31 ± 50.32	68.94^b^	43.86–119.60	1.57 ± 1.50	1.19^e^	0.89–1.68	14.67 ± 10.70	8.00	5.50–25.00	30.33 ± 14.50	28.50	20.75–40.00
VI	73.96 ± 27.86	68.31^c^	56.95–88.94	1.23 ± 0.54	1.14^e^	0.83–1.68	21.11 ± 16.82	17.00	5.00–38.00	32.43 ± 20.11	29.00	15.00–56.00
VII	47.72 ± 18.75	42.07^d^	33.09–57.51	0.97 ± 0.45	0.89^f^	0.61–1.31	14.14 ± 13.57	11.00	7.00–13.00	31.67 ± 10.01	34.50^g^	25.75–38.50
*p*-value	/	0.000	/	/	0.000	/	/	0.919	/	/	0.015	/

*p-value, Kruskal–Wallis one-way analysis of variance; ^a^p < 0.05, compared with group I (Tamhane-adjusted t-test); ^b^p < 0.01, compared with groups II and III (Tamhane-adjusted t-test); ^c^p < 0.05, compared with groups I–III; ^d^p < 0.05, compared with groups I–IV and VI; ^e^p < 0.05, compared with groups II and III (Bonferroni-adjusted t-test); ^f^p < 0.01, compared with groups I–III (Bonferroni-adjusted t-test); ^g^p < 0.05, compared with group III (Bonferroni-adjusted t-test). B81-cVEMP, B81 bone vibrator-induced cervical vestibular-evoked myogenic potential; IAR, interaural asymmetry ratio; IQR, interquartile range; SD, Standard deviation*.

### Response Rate for B81-oVEMP

Using B81 stimuli, the response rate of oVEMP for all subjects was 82% (115/140). The response rate was 100% in groups I–IV, whereas it was 70% in group V, 65% in group VI, and 40% in group VII, exhibiting significant differences between groups I–IV and V–VII (*p* = 0.02, *p* = 0.008, *p* = 0.000, *p* < 0.05, Fisher's exact test; Table 4). There were no statistically significant differences among groups V, VI, and VII (V vs. VI: *p* = 0.741; V vs. VII: *p* = 0.111; VI vs VII: *p* = 0.205; *p* > 0.05, chi-square test).

**Table 4:** The response rate and threshold of B81-oVEMP with increasing age.

**Age groups**	**Response rate**	**Threshold (dB peFL)**		
		**Mean ±SD**	**Median**	**IQR**
I	100% (20/20)	116.00 ± 5.40	114.50	114.50–119.50
II	100% (20/20)	119.75 ± 6.17	119.50	114.50–124.50
III	100% (20/20)	120.00 ± 4.56	119.50	119.50–124.50
IV	100% (20/20)	128.00 ± 4.32	129.50^b^	124.50–129.50
V	70% (14/20)^a^	127.36 ± 5.79	127.00^b^	123.25–134.50
VI	65% (13/20)^a^	129.88 ± 4.31	129.50^b^	124.50–134.50
VII	40% (8/20)^a^	129.50 ± 3.78	129.50^b^	129.50–134.50
*p*-value	0.001	/	0.000	/

*p-value, chi-square test and Kruskal–Wallis one-way analysis of variance; ^a^p < 0.05, Fisher's exact test, compared with groups I–IV; ^b^p < 0.01, Bonferroni-adjusted t-test, compared with groups I–III; B81-oVEMP, B81 bone vibrator-induced ocular vestibular-evoked myogenic potential; IQR, interquartile range; peFL, peak force level; SD, Standard deviation*.

### Threshold of B81-oVEMP

**Figure 4:**
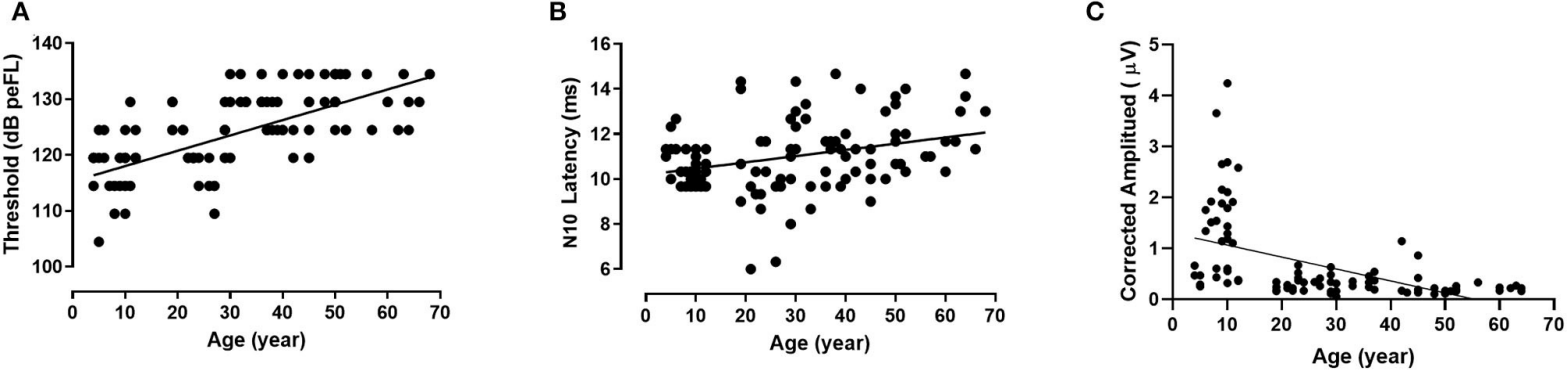
Relationship between age and individual B81-oVEMP parameters (linear regression). The threshold (r^2^ = 0.470, *p* = 0.000, *p* < 0.01) **(A)** and N10 latency (r^2^ = 0.107, *p* = 0.000, *p* < 0.01) **(B)** of B81-oVEMP are positively correlated with age, whereas the corrected amplitude (r^2^ = 0.266, *p* = 0.000, *p* < 0.01) **(C)** is negatively correlated with age.

The thresholds of B81-oVEMP exhibited significant differences between groups I–III and IV–VII (*p* = 0.000, *p* < 0.01, Bonferroni-adjusted *t*-test; Table 4). There was no statistically significant difference between the other groups. Spearman's correlation analysis revealed a significant positive correlation between age and the threshold (r = 0.768, *p* = 0.000, *p* < 0.01). Figure 4A shows that the linear regression curve depicts the increase of B81-oVEMP threshold with age (y = 0.2744x + 115.3).

### N10 and P15 Latencies and N10-P15 Interval of B81-oVEMP

The descriptive statistics (mean, standard deviation, median, and IQR) of latencies and intervals across the age groups are shown in Table 5. There were significant differences in N10 latency, P15 latency, and N10-P15 interval among the seven groups (*p* = 0.000, *p* = 0.001, *p* = 0.005, *p* < 0.01, Kruskal–Wallis test; Table 5). Groups IV and VI showed significantly longer N10 latencies than group III (*p* = 0.007, *p* = 0.004, *p* < 0.01, Bonferroni-adjusted *t*-test). Group VII showed significantly longer N10 latencies than groups II and III (*p* = 0.018, *p* = 0.001, *p* < 0.05, Bonferroni-adjusted *t*-test). For the P15 latency, group III showed significantly shorter latencies than groups IV and VI (*p* = 0.004, *p* = 0.000, *p* < 0.01, Bonferroni-adjusted *t*-test). For the N10-P15 interval, group III showed significantly shorter latencies than group I (*p* = 0.041, *p* < 0.05, Tamhane-adjusted *t*-test), and group VII showed significantly shorter latencies than groups I, II, and VI (*p* = 0.007, *p* = 0.019, *p* = 0.039, *p* < 0.05, Tamhane-adjusted *t*-test). Except for the above-mentioned results, there was no significant difference between the age groups for the N10 latency, P15 latency, and N10-P15 interval. Analyses between the age factor and latencies revealed a significant positive correlation between age and the N10 latency (r = 0.334, *p* = 0.009, *p* < 0.01, Spearman's correlation analysis) but not between age and the P15 latency and N10-P15 interval. The changes in the latencies of B81-oVEMP in each age group are shown in Figure 2D. Figure 4B shows the relationship between age and the N10 latency of B81-oVEMP using a linear regression curve (y = 0.02762x + 10.19).

**Table 5:** The latencies and N10-P15 interval of B81-oVEMP with increasing age.

**Age groups**	**N10 latency (ms)**			**P15 latency (ms)**			**N10-P15 interval (ms)**		
	**Mean ±SD**	**Median**	**IQR**	**Mean ±SD**	**Median**	**IQR**	**Mean ±SD**	**Median**	**IQR**
I	10.77 ± 0.86	10.66	10.00–11.33	16.12 ± 0.84	15.83	15.67–16.91	5.35 ± 0.81	5.34	5.00–6.00
II	10.68 ± 1.32	10.33	10.00–10.92	16.10 ± 1.56	16.00	15.33–16.92	5.42 ± 1.26	5.67	4.08–6.25
III	9.86 ± 1.70	10.00	9.33–11.25	14.03 ± 2.39	14.17^c^	12.42–15.50	4.17 ± 1.38	4.17^d^	3.00–5.25
IV	11.58 ± 1.54	11.50^a^	10.58–12.59	16.35 ± 2.11	16.67	14.58–18.00	4.77 ± 1.40	4.84	3.75–5.92
V	11.05 ± 1.30	10.84	10.00–11.50	15.67 ± 1.50	16.00	14.83–17.00	4.62 ± 1.25	5.00	3.25–5.67
VI	11.77 ± 1.23	11.67^a^	10.67–12.67	17.05 ± 1.26	17.33	15.67–18.00	5.28 ± 0.80	5.33	4.50–6.00
VII	12.42 ± 1.41	12.34^b^	11.42–13.50	16.54 ± 1.80	16.50	15.17–18.34	4.12 ± 0.73	4.00^e^	3.42–4.83
*p*-value	/	0.000	/	/	0.001	/	/	0.005	/

*p-value, Kruskal–Wallis one-way analysis of variance; ^a^p < 0.01, compared with group III (Bonferroni-adjusted t-test); ^b^p < 0.05, compared with groups II and III (Bonferroni-adjusted t-test); ^c^p < 0.01, compared with groups IV and VI (Bonferroni-adjusted t-test); ^d^p < 0.05, compared with group I (Tamhane-adjusted t-test); ^e^p < 0.05, compared with groups I, II, and VI (Tamhane-adjusted t-test). B81-oVEMP, B81 bone vibrator-induced ocular vestibular-evoked myogenic potential; IQR, interquartile range; SD, Standard deviation*.

### Raw Amplitudes, Corrected Amplitudes, Raw IAR, and Corrected IAR of B81-oVEMP

There was a significant difference in the corrected amplitudes between the different age groups (*p* = 0.000, *p* < 0.01), but no significant difference was found in the raw amplitudes, raw IAR, and corrected IAR (*p* = 0.320, *p* = 0.738, *p* = 0.218, *p* > 0.05, Kruskal–Wallis test; Table 6). The corrected amplitudes of groups III, IV, VI, and VII were smaller than that of group I (*p* = 0.000, *p* < 0.01, Tamhane-adjusted *t*-test), those of groups IV, VI, and VII were smaller than that of group II (*p* = 0.000, *p* < 0.01, Tamhane-adjusted *t*-test), and that of group VI was smaller than that of group III (*p* = 0.005, *p* < 0.01, Tamhane-adjusted *t*-test). The corrected amplitudes of B81-oVEMP were significantly correlated with age (r = −0.683, *p* = 0.000, *p* < 0.01, Spearman's correlation analysis). Figure 4C shows the negative correlation between age and corrected amplitudes of B81-oVEMP using a linear regression curve (y = −0.02343x + 1.297).

**Table 6:** Raw amplitudes, corrected amplitudes, raw IAR, and corrected IAR of B81-oVEMP with increasing age.

**Age groups**	**Raw amplitudes (μV)**			**Corrected amplitudes**			**Raw IAR (%)**			**Corrected IAR (%)**		
	**Mean ±SD**	**Median**	**IQR**	**Mean ±SD**	**Median**	**IQR**	**Mean ±SD**	**Median**	**IQR**	**Mean ±SD**	**Median**	**IQR**
I	9.16 ± 6.77	6.71	3.05–11.46	1.29 ± 0.94	1.24	0.47–1.89	18.70 ± 16.53	15.00	4.00–34.00	21.00 ± 10.02	17.00	13.00–28.00
II	10.40 ± 6.23	8.99	4.70–14.39	1.19 ± 1.09	0.85	0.34–1.88	22.90 ± 16.94	22.00	13.00–25.50	36.70 ± 28.34	29.50	9.75–68.00
III	9.80 ± 5.22	7.60	6.66–10.19	0.34 ± 0.15	0.34^a^	0.22–0.41	16.70 ± 14.36	12.00	7.50–25.25	18.40 ± 8.67	17.00	13.00–24.25
IV	7.82 ± 4.13	6.85	4.63–10.17	0.27 ± 0.14	0.29^a, b^	0.18–0.35	19.20 ± 11.56	18.00	10.75–27.75	15.57 ± 12.45	14.00	6.00–31.00
V	11.68 ± 9.21	7.87	6.08–17.66	0.37 ± 0.37	0.19	0.13–0.64	30.00 ± 18.57	23.50	17.50–51.75	41.25 ± 23.27	36.00	23.00–64.75
VI	6.47 ± 2.81	5.56	4.50–8.33	0.17 ± 0.07	0.16^a, b, c^	0.11–0.20	22.50 ± 15.70	15.00	14.00–38.50	21.33 ± 3.22	20.00	19.00–/
VII	9.15 ± 3.79	8.38	6.40–10.27	0.21 ± 0.04	0.22^a, b^	0.17–0.24	17.00 ± 18.39	17.00	4.00–/	14.50 ± 0.71	14.50	14.00–/
*p*-value	/	0.320	/	/	0.000	/	/	0.738	/	/	0.218	/

*p-value, Kruskal–Wallis one-way analysis of variance; ^a^p < 0.01, compared with group I (Tamhane-adjusted t-test); ^b^p < 0.05, compared with group II (Tamhane-adjusted t-test); ^c^p < 0.01, compared with group III (Tamhane-adjusted t-test); B81-oVEMP, B81 bone vibrator-induced ocular vestibular-evoked myogenic potential; IAR, interaural asymmetry ratio; IQR, interquartile range; SD, Standard deviation*.

## Discussion

The VEMP test elicited by ACS is a classical method that is widely employed in clinical settings. Compared to the ACS, BCV has some advantages that overcome the limitations of ACS in the VEMP test. Therefore, it is indispensable to establish the normal range of BCV-induced VEMP for different age groups. Several previous studies have reported the influence of age on oVEMP and cVEMP parameters (17, 24, 25, 27–31). Most studies showed a significant decline in the response rates, prolongation of latencies, and reduction of oVEMP and cVEMP amplitudes with increasing age (24, 33). The association of cVEMP with age has been studied more extensively than that of oVEMP with age. Besides, VEMP parameters are usually affected by position, stimulating method, and setting of the output and record. The results of the studies are not entirely consistent. This study used a Radioear B81 bone vibrator to evoke VEMPs on the mastoids and observed the changes in B81-VEMP parameters with increasing age.

### Effects of Age on the Parameters of B81-cVEMP

The total response rate of B81-cVEMP was 95% (133/140) in our study. The result was similar to that reported by Patterson et al. (92%, 144/156), who used the B81 bone vibrator (17), and Rosengren et al. (93%, 113/122), who used B71 bone vibrator (28). In all the studies, the bone vibrator was applied on the mastoid; however, we used the sitting position, while Patterson et al. and Rosengren et al. used the reclined position. Therefore, both the sitting and reclined positions are suitable for the cVEMP test. In this study, the response rate of B81-cVEMP was 100% up to the age group of 40–49 years and declined to 75% in the group of individuals older than 60 years. The results revealed an increase in threshold values and a decrease in raw and corrected amplitudes in individuals ≥30 years. In addition, the study showed prolongation of the B81-cVEMP P13 and N23 latencies with increasing age.

The reduction in the response rate and both raw and corrected amplitudes with advancing age has been consistently reported across studies. It might be related to the morphological changes and reduced saccular hair cells and neuroepithelia in Scarpa's ganglion and the brainstem vestibular nuclei (31). The prolongation in the P13 and N23 latencies of B81-cVEMP with age can also be explained by age-related changes in the otolith-cervical reflexes based on the alterations in the processing of otolithic signals by the central nervous systems.

### Effects of Age on the Parameters of B81-oVEMP

In the present study, the total response rate of B81-oVEMP was 82% (115/140). Our results were lower than those reported by Patterson et al. (89%, 139/156), who used the B81 bone vibrator (17), but higher than that of Rosengren et al. (65%, 79/122), who used B71 bone vibrator (28). The main difference between the studies was that we placed the vibrator on the mastoid. Rosengren et al. (3) showed that slight displacement of the B81 or B71 vibrator on the mastoid could result in the absence or reduced responses when recording the VEMPs, which was also shown in our VEMP test recording. Another reason may be that Patterson et al. (17) increased the stimulation intensity to 75 dB nHL (138 dB peFL) when oVEMP could not be elicited with 70 dB nHL (136 dB peFL) stimulation. However, in our study, we calibrated the B81 vibrator using artificial mastoid 4,930, and the output was distorted when it was above 65 dB nHL. Hence, we used the maximum stimulus intensity of 65 dB nHL (134.5 dB peFL). It should be noted that the response rates using the B81 outperformed those using the B71, which cannot be attributed to output. This equals the force output of the B81 when the frequency is 500 Hz (16, 17, 28). We believe that forehead stimulation may be the best location for the oVEMP response instead of the mastoid (17, 28, 32). Our results showed that the response rate of B81-oVEMP was 100% up to the age group of 40–49 years and progressively declined afterward to only 40% in the group of individuals ≥60 years. These findings agree with those reported in the literature (14). However, these findings contradicted those reported by a previous study, which showed 100% response rates in both the younger and older groups (12). This may be attributed to the different stimulation conditions. Iwasaki et al. used a Brüel and Kjaer 4810 Mini-shaker or a light tap with a tendon hammer in the middle at the hairline to provide BCV stimuli.

Few studies have explored the effect of age on the oVEMP threshold. Our study showed that the threshold of B81-oVEMP gradually increased with age. A morphological study of the vestibular neuroepithelium revealed that degenerative changes in the vestibular organs occurred after the age of 40 years. Approximately, 20% loss of the hair cell population was noted in the utricular macula with increasing age, consistent with a significant decline in the response rates and increase in the threshold of B81-oVEMP in individuals ≥40 years (14, 20). In addition to age-related changes in the vestibular hair cells, depletion of the vestibular ganglion cells and vestibular afferent neurons are also noted during the aging process. Richter et al. reported a decrease in the number of vestibular hair cells after the age of 20 years and a decrease in vestibular ganglia after the age of 50 years (21). Merchant also reported a gradual loss in vestibular hair cells in all the end organs (three cristae and two maculae) with increasing age (23). A previous study identified significant age-related loss of neurons in the medial vestibular nucleus (33), a well-documented VOR relay center. The prolonged N10 latency of B81-oVEMP may degrade the central vestibular processing of otolithic signals with age.

Our results showed that the P15 latency and raw amplitude of B81-oVEMP were not significantly correlated with age, contradicting the results of previous studies (14, 28). However, the corrected amplitude decreased with increasing age. These data indicate the importance of stimulus parameters (testing position, electrode montage position, vibrator position, force, etc.) in the oVEMP test (3, 34).

### Raw IAR and Corrected IAR of B81-cVEMP and B81-oVEMP

For B81-cVEMP and B81-oVEMP, our results revealed no correlation between the raw IAR and corrected IAR with increasing age. These results are consistent with previous reports (14, 25, 27). The lack of change in the IAR with advancing age could be attributed to the bilateral and usually symmetrical nature of the aging process, which has been reported most often in the studies conducted on the effect of aging on hearing and balance (31).

We also observed the raw IAR and corrected IAR in each group. The corrected amplitude did not reduce the IAR values in our study. Instead, the maximum value of the corrected IAR increased by using the corrected amplitude in most age groups, being more apparent for B81-cVEMP.

During VEMP testing, we found that some subjects had a reduced IAR, but the IAR of other subjects increased using the corrected amplitude. McCaslin et al. found similar results. However, they suggested that the cVEMP test is best performed using optimal EMG activation with amplitude normalization techniques (35). Lee et al. reported that rectified data can produce a more reliable IAR and may help diagnose some vestibular disorders according to amplitude-associated parameters (36). As we only included 10 subjects in each group in this study, the results of our study need to be further explored.

### The Response Rate of B81-oVEMP Decreases More Significantly With Age

This study showed that when patients were older than 60 years, the response rate of B81-cVEMP was 75%, while that of B81-oVEMP was only 40%. B81-oVEMP is an excitatory postsynaptic potential that is not affected by muscle contraction. Still, the recording potential is small, and many interference factors, such as stimulus artifacts, electromagnetic interference, head size, stimulation location, and lower eyelid thickness, could affect its record (37). Besides, the B81 bone vibrator placed on the mastoid typically produces good cVEMP but poorer oVEMP because forehead stimulation may be the best location for the oVEMP response (17, 28). Therefore, when recording oVEMP, the influencing factors need to be carefully controlled. The disappearance of the waveform does not necessarily indicate utricular dysfunction and should be considered comprehensively.

### Limitations

Finally, several limitations to the present study should be considered. First, BCV stimulations have several advantages compared to ACS stimulation. BCV stimulations can be used in patients with conductive hearing loss, decrease the sound exposure in children with small external auditory canal volumes, and produce large oVEMP responses. But BCV stimulation activates the utricle and saccule of both ears, rendering their interpretation more difficult in patients with unilateral vestibular loss (3). Second, BCV stimulation is less sensitive and specific than ACS stimulation when diagnosing third-window diseases (38). Third, the B81 vibrator was placed on the mastoid because it is difficult to attach it firmly to the forehead. VEMPs are sometimes sensitive to small variations in vibrator placement. The vibrator locations should be moved slightly if B81-VEMPs are very small or absent (3). Furthermore, based on a small sample size in this study, the results of patients with vestibular diseases using different BCV stimulations are needed for future research.

## Conclusion

Age significantly affects B81-cVEMP and B81-oVEMP responses, particularly the response rate of B81-oVEMP. Hence, we suggest establishing reference values according to different age groups when evaluating the VEMP responses in patients with vestibular diseases.

## Data Availability Statement

The original contributions presented in the study are included in the article/supplementary material, further inquiries can be directed to the corresponding author/s.

## Ethics Statement

The studies involving human participants were reviewed and approved by Xi'an Jiaotong University. Written informed consent to participate in this study was provided by the participants' legal guardian/next of kin. Written informed consent was obtained from the individual(s) for the publication of any potentially identifiable images or data included in this article.

## Author Contributions

YZ and ZC wrote the manuscript. PR, QZ, and YJ provided the idea for the study, reviewed, and edited the manuscript. JY reviewed and edited the manuscript. HZ, JS, and QW collected the clinical data. All authors contributed to the article and approved the submitted version.

## Funding

This work was supported by the National Natural Science Foundation of China (Grant Nos: 81970891, 82171137, and 81971766) and the Key International Cooperation Project of Shaanxi Province (Grant No: 2020-KWZ-019) and the Science and Technology Project of Shanghai Science and Technology Commission (Grant No: 21S31900600).

## Conflict of Interest

The authors declare that the research was conducted in the absence of any commercial or financial relationships that could be construed as a potential conflict of interest.

## Publisher's Note

All claims expressed in this article are solely those of the authors and do not necessarily represent those of their affiliated organizations, or those of the publisher, the editors and the reviewers. Any product that may be evaluated in this article, or claim that may be made by its manufacturer, is not guaranteed or endorsed by the publisher.

## Abbreviations

B81-cVEMP: B81 bone vibrator-induced cervical vestibular-evoked myogenic potentials
B81-oVEMP: B81 bone vibrator-induced ocular vestibular-evoked myogenic potentials
VEMP: vestibular-evoked myogenic potential
SCM: sternocleidomastoid
cVEMP: cervical VEMP
oVEMP: ocular VEMP
VOR: vestibulo-ocular reflex
ACS: air-conducted sound
BCV: bone-conducted vibration
peFL: peak force level
EMG: electromyogram.

## References

[1] Curthoys IS Grant JW Burgess AM Pastras CJ Brown DJ Manzari L. Otolithic receptor mechanisms for vestibular-evoked myogenic potentials: a review. Front Neurol. (2018) 9:e366. 10.3389/fneur.2018.00366 29887827 PMC5980960

[2] Curthoys IS Grant JW Pastras CJ Brown DJ Burgess AM Brichta AM. review of mechanical and synaptic processes in otolith transduction of sound and vibration for clinical VEMP testing. J Neurophysiol. (2019) 122:259–76. 10.1152/jn.00031.2019 31042414

[3] Rosengren SM Colebatch JG Young AS Govender S Welgampola MS. Vestibular evoked myogenic potentials in practice: methods, pitfalls and clinical applications. Clin Neurophysiol Pract. (2019) 4:47–68. 10.1016/j.cnp.2019.01.005 30949613 PMC6430081

[4] Colebatch JG Halmagyi GM. Vestibular evoked potentials in human neck muscles before and after unilateral vestibular deafferentation. Neurology. (1992) 42:1635–6. 10.1212/WNL.42.8.1635 1641165

[5] Colebatch JG Halmagyi GM Skuse NF. Myogenic potentials generated by a click-evoked vestibulocollic reflex. J Neurol Neurosurg Psychiatry. (1994) 57:190–7. 10.1136/jnnp.57.2.190 8126503 PMC1072448

[6] Rosengren SM McAngus Todd NP Colebatch JG. Vestibular-evoked extraocular potentials produced by stimulation with bone-conducted sound. Clin Neurophysiol. (2005) 116:1938–48. 10.1016/j.clinph.2005.03.019 15979939

[7] Rosengren SM Welgampola MS Colebatch JG. Vestibular evoked myogenic potentials: past, present and future. Clin Neurophysiol. (2010) 121:636–51. 10.1016/j.clinph.2009.10.016 20080441

[8] Curthoys IS. A critical review of the neurophysiological evidence underlying clinical vestibular testing using sound, vibration and galvanic stimuli. Clin Neurophysiol. (2010) 121:132–44. 10.1016/j.clinph.2009.09.027 19897412

[9] Halmagyi GM Yavor RA Colebatch JG. Tapping the head activates the vestibular system: a new use for the clinical reflex hammer. Neurology. (1995) 45:1927–9. 10.1212/WNL.45.10.1927 7477996

[10] Han P Zhang R Chen Z Gao Y Cheng Y Zhang Q. Evaluation of ocular and cervical vestibular evoked myogenic potentials in a conductive hearing loss model. J Otol. (2016) 11:192–7. 10.1016/j.joto.2016.12.002 29937829 PMC6002615

[11] Iwasaki S McGarvie LA Halmagyi GM Burgess AM Kim J Colebatch JG. Head taps evoke a crossed vestibulo-ocular reflex. Neurology. (2007) 68:1227–9. 10.1212/01.wnl.0000259064.80564.21 17420408

[12] Iwasaki S Smulders YE Burgess AM McGarvie LA MacDougall HG Halmagyi GM. Ocular vestibular evoked myogenic potentials to bone conducted vibration of the midline forehead at Fz in healthy subjects. Clin Neurophysiol. (2008) 119:2135–47. 10.1016/j.clinph.2008.05.028 18639490

[13] Basta D Todt I Ernst A. Characterization of age-related changes in vestibular evoked myogenic potentials. J Vestib Res. (2007) 17:93–8. 10.3233/VES-2007-172-304 18413902

[14] Tseng CL Chou CH Young YH. Aging effect on the ocular vestibular-evoked myogenic potentials. Otol Neurotol. (2010) 31:959–63. 10.1097/MAO.0b013e3181e8fb1a 20601917

[15] Welgampola MS Rosengren SM Halmagyi GM Colebatch JG. Vestibular activation by bone conducted sound. J Neurol Neurosurg Psychiatry. (2003) 74:771–8. 10.1136/jnnp.74.6.771 12754349 PMC1738493

[16] Clinard CG Piker EG Thorne AP Surface EN Anderson AE Beacham VA. Maximum output and low-frequency limitations of B71 and B81 clinical bone vibrators: implications for vestibular evoked potentials. Ear Hear. (2020) 41:847–54. 10.1097/AUD.0000000000000808 31613822

[17] Patterson JN Rodriguez AI Gordon KR Honaker JA Janky KL. Age effects of bone conduction vibration vestibular-evoked myogenic potentials (VEMPs) using B81 and impulse hammer stimuli. Ear Hear. (2021) 42:1328–37. 10.1097/AUD.0000000000001024 33735908 PMC8387331

[18] Jansson KJ Håkansson B Johannsen L Tengstrand T. Electro-acoustic performance of the new bone vibrator radioear B81: a comparison with the conventional radioear B71. Int J Audiol. (2015) 54:334–40. 10.3109/14992027.2014.980521 25519145

[19] Romero DJ Piker EG Thorne A Clinard C. Comparison of bone-conducted cervical VEMPs elicited by B71 and B81 bone vibrators. Ear Hear. (2021) 42:596–605. 10.1097/AUD.0000000000000978 33577217

[20] Rosenhall U. Degenerative patterns in the aging human vestibular neuro-epithelia. Acta Otolaryngol. (1973) 76:208–20. 10.3109/00016487309121501 4543916

[21] Richter E. Quantitative study of human Scarpa's ganglion and vestibular sensory epithelia. Acta Otolaryngol. (1980) 90:199–208. 10.3109/00016488009131716 6258381

[22] Merchant SN Velázquez-Villaseñor L Tsuji K Glynn RJ Wall C III Rauch SD. Temporal bone studies of the human peripheral vestibular system normative vestibular hair cell data. Ann Otol Rhinol Laryngol Suppl. (2000) 181:3–13. 10.1177/00034894001090S502 10821229

[23] Velázquez-Villaseñor L Merchant SN Tsuji K Glynn RJ Wall C III Rauch SD. Temporal bone studies of the human peripheral vestibular system normative Scarpa's ganglion cell data. Ann Otol Rhinol Laryngol Suppl. (2000) 181:14–9. 10.1177/00034894001090S503 10821230

[24] Macambira YKD Carnauba ATL Fernandes LCBC Bueno NB Menezes PD. Aging and wave-component latency delays in oVEMP and cVEMP: a systematic review with meta-analysis. Braz J Otorhinolar. (2017) 83:475–87. 10.1016/j.bjorl.2016.12.006 28237301 PMC9442875

[25] Singh NK Firdose H. Characterizing the impact of advancing age on 500 Hz tone-burst evoked ocular-vestibular evoked myogenic potentials. Eur Arch Otorhinolaryngol. (2021) 278:4259–68. 10.1007/s00405-020-06542-2 33454811

[26] Tseng CC Wang SJ Young YH. Comparison of head elevation versus rotation methods for eliciting cervical vestibular-evoked myogenic potentials via bone-conducted vibration. Int J Audiol. (2013) 52:200–6. 10.3109/14992027.2012.754108 23336671

[27] Welgampola MS Colebatch JG. Vestibulocollic reflexes: normal values and the effect of age. Clin Neurophysiol. (2001) 112:1971–9. 10.1016/S1388-2457(01)00645-9 11682335

[28] Rosengren SM Govender S Colebatch JG. Ocular and cervical vestibular evoked myogenic potentials produced by air- and bone-conducted stimuli: comparative properties and effects of age. Clin Neurophysiol. (2011) 122:2282–9. 10.1016/j.clinph.2011.04.001 21550301

[29] Colebatch JG Govender S Rosengren SM. Two distinct patterns of VEMP changes with age. Clin Neurophysiol. (2013) 124:2066–8. 10.1016/j.clinph.2013.04.337 23757380

[30] Su HC Huang TW Young YH Cheng PW. Aging effect on vestibular evoked myogenic potential. Otol Neurotol. (2004) 25:977–80. 10.1097/00129492-200411000-00019 15547429

[31] Singh NK Kashyap RS Supreetha L Sahana V. Characterization of age-related changes in sacculocolic response parameters assessed by cervical vestibular evoked myogenic potentials. Eur Arch Otorhinolaryngol. (2014) 271:1869–77. 10.1007/s00405-013-2672-0 23982670

[32] Rodriguez AI Marler E Fitzpatrick D Creutz T Cannon SA Thomas MLA. Optimization of cervical and ocular vestibular evoked myogenic potential testing using an impulse hammer in adults, adolescents, and children. Otol Neurotol. (2020) 41:817–27. 10.1097/MAO.0000000000002632 32221109 PMC7311239

[33] Tang Y Lopez I Baloh RW. Age-related change of the neuronal number in the human medial vestibular nucleus: a stereological investigation. J Vestib Res. (2001-2002) 11:357–63. 10.3233/VES-2002-11602 12446961

[34] Govender S Colebatch JG. Effects of midline sagittal location on bone-conducted cervical and ocular vestibular evoked myogenic potentials. J Appl Physiol (1985). (2017) 122:1470–84. 10.1152/japplphysiol.01069.2016 28336540

[35] McCaslin DL Jacobson GP Hatton K Fowler AP DeLong AP. The effects of amplitude normalization and EMG targets on cVEMP interaural amplitude asymmetry. Ear Hear. (2013) 34:482–90. 10.1097/AUD.0b013e31827ad792 23361360

[36] Lee KJ Kim MS Son EJ Lim HJ Bang JH Kang JG. The usefulness of rectified VEMP. Clin Exp Otorhinolaryngol. (2008) 1:143–7. 10.3342/ceo.2008.1.3.143 19434246 PMC2671751

[37] Curthoys IS Manzari L Smulders YE Burgess AM. A review of the scientific basis and practical application of a new test of utricular function – ocular vestibular-evoked myogenic potentials to bone-conducted vibration. Acta Otorhinolaryngol Ital. (2009) 29:179–86. 20161874 PMC2816364

[38] Janky KL Nguyen KD Welgampola M Zuniga MG Carey JP. Air-conducted oVEMPs provide the best separation between intact and superior canal dehiscent labyrinths. Otol Neurotol. (2013) 34:127–34. 10.1097/MAO.0b013e318271c32a 23151775 PMC3621128

